# Comparison of cheese with and without *Propionibacterium freudenreichii* subsp. *shermanii* LMGT 2951 on off-season muscle strength and VO_2_ max development in Nordic skiers: a randomized clinical trial

**DOI:** 10.1080/15502783.2025.2566373

**Published:** 2025-09-30

**Authors:** Helge Einar Lundberg, Stig Larsen, Therese Fostervold Mathisen, Jorunn Sundgot-Borgen

**Affiliations:** a Norwegian School of Sports Sciences, Department of Sports Medicine, Oslo, Norway; b Meddoc AS, Hvamstubben 14, Skjetten, Norway; c Østfold University College, Department of Health and Welfare, Halden, Norway

**Keywords:** REDs, performance, probiotics, osteocalcin, propionibacterium freudenreichii, cheese

## Abstract

**Background and objective:**

Nordic skiers represent a low-impact, weight-sensitive sport and may be inclined to engage in weight-controlling behavior, hence increasing their risk for low energy availability and impairment in health and performance. Jarlsberg cheese (J) is rich in vitamin K₂ from fermentation by Propionibacterium freudenreichii subsp. shermanii LMGT 2951 (Pf) and lactic acid bacteria and increases the osteocalcin (OC) level. Vitamin K is essential for activating OC, which is described to be associated positively with muscle strength, whereas propionic acid bacteria are found to enhance endurance. Except for Pf and its by-products, J and Norvegia (N) cheeses have almost identical nutrient content. The objective of this study was to compare the effects of Jarlsberg and N as potential supplements to training on muscle strength (MS), lean body mass (LBM) and VO_2_ max.

**Methods:**

Thirty female and 30 male Nordic skiers were block randomized 1:1 to either J or N and studied during a 24-week offseason training schedule. The Norvegia group served as a control group. Females and males were treated with 75 and 90 grams/day, respectively. MS by seated pulldown and half-squat, LBM by DXA, and VO_2_ max were measured at baseline and after the 24 weeks off-season period. Dietary intake and training hours were registered and monitored at baseline and every eight weeks during the study.

**Results:**

MS significantly increased in both groups after 24 weeks (*p* < 0.01). The mean increase difference in MS-upper body favored Jarlsberg by 0.95 kg (95% CI: −0.02–1.46), approaching significance (*p* = 0.06). No significant difference was found between groups in MS-lower body. LBM and VO2-max both increased significantly in each group (*p* ≤ 0.05), with no notable differences between groups. Correlation analysis identified LBM as the dominant outcome variable at both baseline and 24 weeks. Endurance training was the dominant input variable and correlated significantly positively both multiply and partially to LBM, (*p* = 0.04, *p* = 0.02), respectively. At baseline and 24-weeks sex, endurance training along with OC-level explained 65% and 68% of the LBM variations, respectively.

**Conclusion:**

Both Jarlsberg cheese, characterized by its unique Propionibacterium freudenreichii content, and Norvegia cheese, when combined with off-season training, led to increases in LBM, VO₂ max, and MS, with no significant differences observed between groups. However, there was a trend toward greater improvements in MS in the Jarlsberg group. Overall, increases in LBM through structured training appear to be a key driver of gains in both muscle strength and aerobic capacity.

**Protocol number:**

XCS-Jarlsberg/IIA

**ClinicalTrial.gov:**

NCT06688032

## 1. Introduction

Relative energy deficiency in sport (REDs) is a syndrome of impaired physiological and psychological functioning experienced by athletes upon exposure to problematic low energy availability (LEA) [1]. Problematic LEA results in several clinical symptoms like impaired endocrine function and decreased estrogen in females, testosterone in males and reduced muscle mass [1]. Because low body mass can enhance work economy in cross-country and biathlon skiing, many skiers practice restrictive eating behavior to improve performance [2]. As such, these athletes are at increased risk for problematic LEA and REDs.

Osteocalcin (OC), a peptide specifically produced by osteoblasts, plays a crucial role in bone calcification. Additionally, it is associated with several potential properties that may beneficially counteract many of the negative effects of low energy availability (LEA) [3,4]. OC has been reported to have a muscle-protective effect, maintaining muscular strength during weight loss, and serum OC levels correlate positively with skeletal muscle mass in postmenopausal osteoporotic women [4,5]. Administration of exogenous OC to older OC-gene depleted mice increases muscle mass and the myofiber cross-sectional area, suggesting its potential for preventing muscle wasting [6]. OC is proposed to stimulate testosterone production in the testes [7,8]. However, varying study measurements of OC complicate uniform data extraction [7,9,10]. Carboxylated osteocalcin (cOC) is crucial for bone health, with recent studies showing that cOC, rather than undercarboxylated OC (ucOC), protects muscle mass during weight loss in healthy, obese men, mediating bone-muscle crosstalk [4,5].

**Figure 1.**
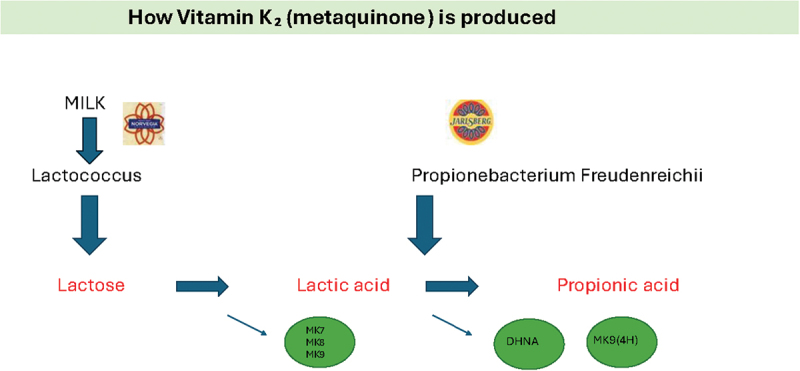
How Norvegia and Jarlsberg cheeses are made from the same milk. The fermentation of both cheeses starts with a starter culture of lactic acid bacteria. The medium chained K_2_- vitamers MK-7, MK-8 and MK-9 are then produced. In the Jarlsberg curd propione bacterium freudenreichii ferments of lactic acid to propionic acid. The by products are 1,4- dihydroxy 2- naphthoic acid (DHNA) and the long chained, side chained K_2_-vitamer MK-9(4 H). Jarlsberg cheese contains twice as much vitamin K_2_ as Norvegia cheese.

Vitamin K is essential for the carboxylation ucOC to cOC [11–13]. In the Western diet, fermented dairy products like cheese are the best sources of long-chained K₂-vitamers (menaquinones) which have proven better than the short-chained and vitamin K₁ to carboxylate ucOC [12,14]. The commercially available Jarlsberg cheese (J) and Norvegia cheese (N) are both rich in long-chained K₂-vitamers, produced by bacterial fermentation. Made from the same milk, J is produced by a mixed strain mesophilic starter culture of lactic acid bacteria and Propionibacterium freudenreichii subsp. shermanii LMGT 2951, whereas N is only by the starter culture (Figure 1). Mature J cheese is reported to contain more than ~10^8^ live colony forming units (CFU) of Pf per gram even after storage. In addition to MK-9(4 H), this process also produces 1,4- dihydroxy 2- naphthoic acid (DHNA), which boosts total OC (tOC) [15]. Systemic effects, measured by osteocalcin production, were evident after 3 weeks of daily Jarlsberg consumption [16]. Of relevance, systemic lactate is capable of crossing the epithelial barrier to the gut lumen [17]. It has been proposed that a high-lactate gut environment provides a selective advantage for colonization by propionic acid-forming bacteria like Pf [17]. Pf is tolerant to digestive stress and maintains its metabolic activity in the human digestive tract [18]. It can metabolize lactate as its sole energy source, forming propionate, and can consume intestinal lactate. Serum lactate is absorbed by the gut, where Pf converts it to propionate, which can then reenter the bloodstream and be utilized by the body. Increasing gut propionate through intrarectal installation or by supplementing bacteria capable of converting lactate to propionate, has been shown to increase treadmill run time performance in mice cross-over studies [17].

Propionate has been shown to increase heart rate, VO2 max, and blood pressure in mice, as well as increase resting energy expenditure and lipid oxidation in fasted humans [17]. It is conceivable that feeding Pf to athletes will have similar effects, enhancing endurance. However, the similar results in both cheese groups left the causes unclear [18].

A previous dose-response study on cheese and bone markers showed an increase in VO₂ max and muscle strength (MS) indicating an anabolic effect [19]. However, as there were similar results in the two cheese groups in the present study, the causes of these changes remain unclear. This controlled clinical trial aimed to compare the effects of J and N on MS, VO₂-max, and lean body mass (LBM). The Norvegia-group (N-group) served as a control group.

The hypothesis was that J because of its Pf content would result in better VO₂ max than N group. Additionally, it was hypothesized that J, with its superior content of vitamin K combined with training, would increase MS strength more effectively compared to the N.

## 2. Methods

### 2.1. Study population

The study population consisted of female and male cross-country and biathlon skiers at tier 3–4 [20]. Exclusion criteria included eating disorders (ED), diseases affecting oral nutrition uptake, milk protein allergy, verified cancer, pregnancy, systemic treatment with corticosteroids in the last three weeks prior to enrollment, or participation in another clinical trial.

### 2.2. Study sample

To obtain a significant level of 0.05; a power of 90% and clinically relevant difference (CRD) between groups of one-time standard deviation (SD) related to bone markers, a minimum of 23 participants were required in each group to complete the studies. The study sample consisted of 30 female and 30 male athletes, recruited by advertisement through social media, snowball recruitment, and internally at Norwegian School of Sports Sciences (NSSS). Related to the bone health section of this project, females were included from 17 years of age due to earlier skeletal maturation in females, and males from 18 years of age. All participants signed the informed consent form before inclusion. One participant from each treatment group dropped out during the study (Table 1). One female athlete dropped out from the N-group due to an injury, and one male from the J-group because he ended his sports career. They both attended at baseline. The treatment groups were comparable on all recorded demographic data, concomitant diseases and asthma disorders (Table 1).

**Table 1.** Baseline demographics and clinical characteristics. The results are expressed by mean values and total range.

Variables	Jarlsberg group		Norvegia group		Total	
	Female n = 15	Male n = 14	Female n = 14	Male n = 15	Jarlsberg n = 29	Norvegia n = 29
Age (year)	20	22	20	20	21	20
	17–27	18–30	17–26	18–25	18–30	17–26
BMI (kg/m^2^)	21.9	22.7	22.2	22.0	22.3	22.1
	19.2–24.8	18.3–26.2	18.7–25.5	18.7–26.0	18.3–26	18.7–26.0
Number of menstruation/years	11		11		11	11
	6–12		0–12		6–12	0–12
Testosterone (nmol/L)		21.8		20.3	21.8	20.3
		12–32		14–27	12–32	14–27

### 2.3. Study design and randomization

The study was performed as a randomized clinical trial with stratified parallel group design at NSSS. The random allocation sequence was generated by SL, and it was blinded to the primary investigator at baseline. The two cheeses are different in appearance, taste and smell, hence further blinding was not possible. Principally we cannot adjust for an unblinded design, but all participants were eager to follow their training plan, and both cheese intake and physiological variables were monitored. The participants were stratified by sex and equally randomly assigned 1:1 to the J-group and N-group by block randomization with fixed block size of 10. The study was conducted during the off-season, with each participant’s study period lasting 24 weeks, from April 17 to 15 October 2023. This period began approximately three weeks after the competitive season and extended to the start of their most intensive training period.

### 2.4. Clinical procedure

Clinical status, interview and blood samples were performed at baseline and every eight weeks. Measurements of upper and lower body muscle strength and VO_2_-max were performed at baseline and week 24.

### 2.5. Measurements

The set of outcome variables consist of maximum upper body muscle strength (MS_ub_), maximum lower body muscle strength (MS_lb_), VO₂ max and Lean body mass (LBM). The set of input variables consists of training, cOC, tOC, the ratio between procollagen type 1 N-terminal propeptide (P1NP) and cross-linked C-teleopeptide type 1 collagen (CTX) (P1NP/CTX-ratio) and testosterone in men.

MS_ub_ was evaluated by seated pull-down using a Technogym Radiant apparatus, and MS_lb_ by half-squat performed in a Smith machine as described for XCS [21]. VO_2_ max was measured by an incremental VO₂ max test running on a treadmill to volitional exhaustion at a 6° incline and initiated at an individualized speed. The test progressed with increases of 1 km/h every minute until the participant gave hand signal of 0.5 km/h increase or no further increase. The highest continuous VO₂ during a 60 second period was defined as VO₂ max [22]. Several studies at NSSS have been performed with the same protocol during decades, both in elite athletes and adolescents [23,24]. During the study, some participants suffered from intercurrent infections or injuries leading to material reduction in the analysis of MS and VO_2_ max (Table 2). The primary focus when creating the protocol was to compare the impact of the two types of cheese on bone mass [25]. Secondary measures were implemented to control the intervention and monitor the participants’ expected development during the off-season period.

**Table 2.** Comparison of Jarlsberg® and Norvegia cheese in development of muscle strength, lean body mass, VO^2^ and testosterone during 24 weeks of daily cheese intake. The total consists of 29 female and 29 male skiers of which 15 females and 14 males in the Jarlsberg group and 14 females and 15 males in the Norvegia group. The results are expressed by means with SD in brackets and 95% confidence intervals.

Variables	Sex	Baseline		Week 24		Increase from baseline		Cohens d	
		Jarlsberg	Norvegia	Jarlsberg	Norvegia	Jarlsberg	Norvegia	Sex	Total
Muscle strength Upper body (Pull down) (kg)	Female (*n* = 28)	n = 15 27.5 (3.7) 25.5–29.5	n = 13 31.7 (4.3) 29.2–34.3	n = 15 29.5 (3.3) 27.7–31.3	n = 13 32.5 (4.6) 29.7–35.3	n = 15 2.0 (2.4) 0.7–3.3	n = 13 0.8 (1.6) −0.2–1.7	0.60	0.38
	Male (*n* = 29)	n = 14 40.2 (5.8) 36.9–43.5	n = 15 38.3 (4.1) 36.1–40.6	n = 14 42.7 (5.5) 39.5–45.9	n = 15 40.0 (4.2) 37.7–42.3	n = 14 2.5 (3.7) 0.4–4.6	n = 15 1.7 (1.8) 0.7–2.7	0.29	
Muscle strength Lower body (Max squat) (kg)	Female (*n* = 24)	n = 13 112.2(32.4) 92.6–131.7	n = 11 128.6 (20.7) 114.7–142.6	n = 13 129.6 (15.7) 120.1–139.1	n = 11 136.4 (22.6) 121.2–151.5	n = 13 17.5 (33.9) −3.0–37.9	n = 11 7.7 (8.8) 1.8–13.6	0.45	0.11
	Male (*n* = 23)	n = 11 158.6 (23.1) 143.1–174.2	n = 12 148.8 (30.9) 129.1–168.4	n = 11 167.7 (17.1) 156.3–179.2	n = 12 163.8 (34.7) 141.7–185.8	n = 11 9.1 (15.1) −1.1–19.3	n = 12 15.0 (14.0) 6.1–23.9	0.41	
Lean body mass (kg)	Female (*n* = 29)	n = 15 44.71(40.77) 42.47–46.96	n = 14 48.48 (54.8) 45.32–51.6	n = 15 45.30 (37.0) 43.25–47.34	n = 14 49.27 (50.1) 46.38–52.16	n = 15 0.59 (0.94) 0.07–1.11	n = 14 0.79 (1.29) 0.04–1.54	0.18	0.16
	Male (*n* = 29)	n = 14 61.19(59.53) 57.76–64.63	n = 15 58.91 (50.82) 56.10–61.72	n = 14 61.79 (53.4) 58.71–64.87	n = 15 59.7 (50.2) 56.91–62.5	n = 14 0.60 (1.15) −0.07–1.26	n = 15 0.78 (1.34) 0.04–1.52	0.15	
VO^2^ (ml/kg/min)	Female (*n* = 26)	n = 14 57.5 (4.7) 54.8–60.2	n = 12 55.9 (4.0) 53.4–58.4	n = 14 59.9 (5.0) 57.0–62.7	n = 12 58.5 (3.4) 56.4–60.7	n = 14 2.3 (2.1) 1.2–3.5	n = 12 2.6 (2.3) 1.2–4.1	0.14	0.19
	Male (*n* = 27)	n = 12 69.6 (6.4) 65.6–73.7	n = 15 69.7 (4.5) 67.2–72.1	n = 12 72.8 (6.8) 68.5–77.1	n = 15 73.4 (3.7) 71.4–75.5	n = 12 3.1 (3.8) 0.7–5.6	n = 15 3.8 (3.0) 2.1–5.4	0.20	
Testosterone (nmol/L) *^)^	Male (*n* = 28)	n = 13 21.2 (4.9) 18.3–24.2	n = 15 20.3 (3.8) 18.2–22.3	n = 13 23.1 (6.5) 19.4–26.9	n = 15 20.1 (4.3) 17.7–22.4	n = 13 1.9 (6.7) −2.1–6.0	n = 15 −0.2 (4.6) −2.8–2.4		0.72

LBM was measured according to recommended procedure at baseline and final visit using a dual energy X-ray absorptiometry (Lunar iDXA, E’CORE Software, version 14.10.022; GE Healthcare, Madison, WI) at lumbar spine (L1-L4), femoral neck, total hip and total body [26].

Blood samples included serum for analyses of primary and supporting variables. Osteocalcin was analyzed by Vitas laboratories in Oslo, and K₂ at the biochemical laboratory of Norwegian University of Life Sciences [16,27].

### 2.6. Diet registration

The participants were asked not to change their usual diet during the study, except for avoiding cheese other than the cheese provided in the study. A diet registration was performed for four days at baseline, and every eight weeks during the study. The participants registered their dietary intake in an application based on the Norwegian food table [28]. The app used by the athletes was a digital adaptation of the Norwegian food table. The athletes recorded all their food and drink intake according to the food list, and a registered dietitian continuously monitored the recordings and to ensure compliance, consulted individually with each athlete if discrepancies appeared evident. Medin concludes in her doctoral dissertation that although dietary registration methods are uncertain, digital ones are preferable [29]. No macronutrient adjustments were made based on this input.

### 2.7. Training registration

No training intervention was conducted. The participants followed their own cross-country ski-specific training programs, primarily consisting of endurance exercises aimed at both strength and aerobic capacity. Much of the muscular endurance training was derived from ski-specific training methods, such as roller skiing, but also running and cycling. In addition to their diet registration, the skiers registered their weekly hours of strength and endurance training at baseline and every eight weeks during the study.

### 2.8. Clinical intervention

Following determination of optimal cheese/OC-doses (OED), the female participants in both groups received 5 slices of cheese (75 g, and the male participants received 6 slices (90 g). The cheese was delivered to the investigation team by the manufacturer TINE, a partner in the research project [30].

Because Jarlsberg® (and Norvegia®) cheeses are commercially available, the manufacturer’s laboratory regularly analyzes the content of bacteria to ensure the bacterial count is stable, but each batch delivered to the clinical study was not analyzed prior to delivery. The daily Jarlsberg doses contained 75 × 10^8^ and 90 × 10^8^ cfu of *Propionibacterium freudenreichii* subsp. *shermanii* LMGT 2951 to the female and male participants, respectively. This is a general statement. This content has been determined using an agar plate method, which provides the count of viable PF found in Jarlsberg cheese. Sodium lactate agar was used, and the plates were incubated at 30°C for 9 days. The analysis method employed is an internal method used by TINE. They have data indicating that the number of live bacteria remains constant throughout the storage period. The cheese was delivered in several batches, and the participants received new packages of cheese every 4^th^ week.

The daily dose of cheese provides 33 and 40 mcg K_2_-vitamers to female and male participants in the N group, and 64 and 77 mcg to female and male participants in the J-group, respectively. Per 100 g the contents of J are Energy 1458 KJoules (351 Kcal), fat 27 g, protein 27 g, carbohydrates 0 g, vit K 88 µg. The contents of 100 g N are Energy 129 KJoules (344Kcal), fat 26 g, protein 27 g, carbohydrates 0 g, vit K 45.6 µg.

To adjust for any vitamin D deficiency, all participants were provided vitamin D-pearls (Pharma Nord, Denmark) 40 mcg from baseline. The dosage was individually adjusted according to baseline vitamin D status, with the aim of reaching 100 nmol/L during the study. Cheese and vitamin D intake and training breaks due to illness or injuries were recorded every four weeks.

### 2.9. Cheese compliance

In females, there was no significant difference in cheese compliance between groups; 87.3% and 89.5% in the J-group and N-group, respectively. In males, the compliance was significantly better in the N-group by 99.5%, and 88% in the J-group.

### 2.10. Statistical analysis

This study was designed and conducted as an open randomized clinical trial, primarily aimed at comparing the effects of two types of cheese on bone health, using Osteocalcin and bone markers as primary variables [25]. The sample size estimation was calculated with a significance level of 5%, a power of 90%, and a Clinically Relevant Difference (CRD) of 1xSD. Based on this power analysis, significant differences between groups of 1xSD in all unimodal continuously distributed variables should be detectable. However, in all physiological variables recorded in this study, the mean differences between groups were less than 1xSD, rendering them too small to achieve statistical significance.

If we reduce to CRD < 0.2xSD it might be possible to obtain significant difference between groups within both sexes for MS and LBM and in the total material for VO2. However, this will increase the sample size approximately 10 times.

All tests within and between groups were performed two-tailed and differences considered significant with *p* ≤ 0.05. The probability distribution for all the main variables were tested by Shapiro–Wilk test and Kolmogorov–Smirnov test [31,32]. In case of skewed distribution, a logarithmic transformation of the variables was carried out prior to the analysis. The results from the analysis were retransformed for presentation.

The continuously distributed variables were expressed by mean values with 95% confidence interval (CI) [33]. As an index of dispersion, SD was given. Analysis of covariance (ANCOVA) with baseline measurement and sex as a covariate was used for comparison of groups [34]. Changes within groups were performed by Analysis of Variance (ANOVA) [33]. Cohen’s d is included as a measure of effect size [35] (Table 2). The size of the material is too small to perform sub-group analyses to obtain valid results.

Multiple- and partial Pearson correlation model was used to investigate the correlation pattern within the set of outcome variables and between the input and outcome variables [34].

### 2.11. Approvals

The study was approved by the Ethical committee at Norwegian School of Sport Sciences (NSSS) (approval number 273–160323) and was performed in accordance with the research protocol. (Protocol number: XCS-Jarlsberg/IIA, ClinicalTrial.gov: NCT06688032).

## 3. Results

**Figure 2.**
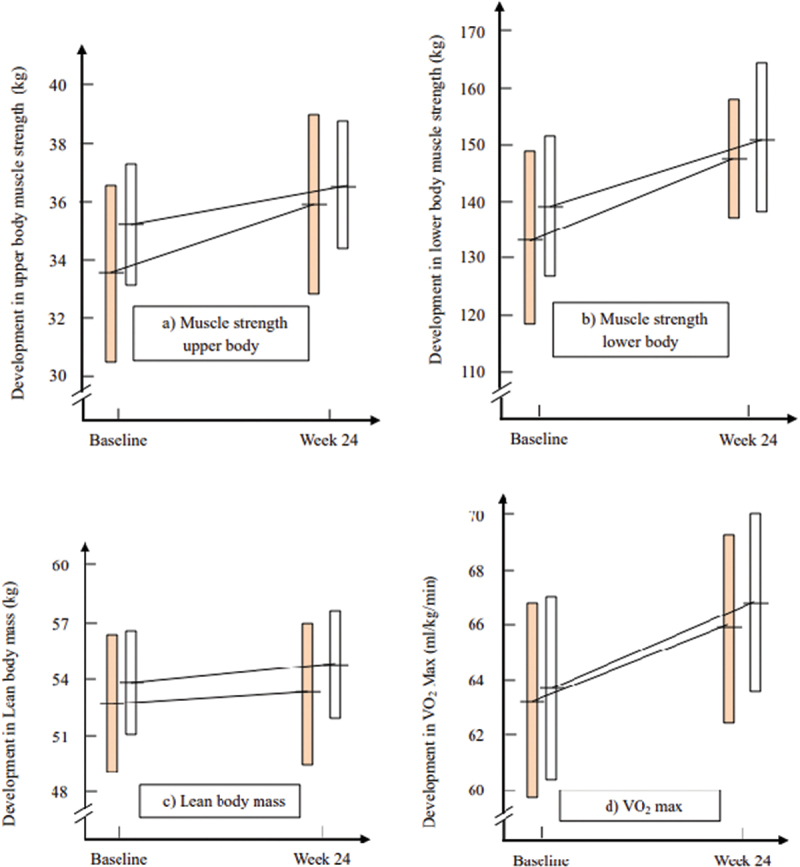
Development in a) Muscle strength upper body (MS_ub_), b) lower body (MS_lb_), c) Lean body mass (LBM) and d)VO_2_ Max from baseline to week-24. The columns express 95% confidence intervals of the mean values indicated by the horizontals line crossing the orange columns 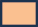 for the Jarlsberg group and white columns 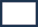 for Norvegia.

MS_ub_ increased from 33.6 kg/BW (95% CI: 30.6–36.7) at baseline to 35.9 kg/BW (95% CI: 32.8–38.9) after 24 weeks in the J-group (*p* < 0.001), and from 35.3 kg/BW (95% CI: 33.2–37.3) to 36.5 kg/BW (95% CI: 34.3–38.7) in the N-group (*p* < 0.001) (Figure 2(a)). The MS_ub_ increase was significant and similar in the two sex-groups for the J-group, but most pronounced and significant for males in the N-group (Table 2). No significant differences between the cheese groups were detected, but the increase among females in the J-group was more pronounced compared to the N-group (*p* = 0.06).

MS_lb_ increased in both cheese-groups without significant differences between groups (Figure 2(b)). MS_lb_ increased from 133.5 kg/BW (95% CI: 118.0–148.9) at baseline to 147.1 kg/BW (95% CI: 136.5–157.7) in the J-group (*p* = 0.02) and from 139.1 kg/BW (95% CI: 127.1–151.2) to 150.7 kg/BW (95% CI: 136.8–164.5) in the N-group (*p* < 0.001). MS_lb_ increased significantly within both sex-groups in the N-group but not separately for neither sex-group in the J-group (Table 2). No significant difference between the J- and N-group was detected.

LBM increased from 52.7 kg (95% CI: 49.0–56.4) at baseline to 53.3 kg (95% CI: 49.6–56.9) after 24 weeks in the J-group (*p* = 0.004) (Figure 2(c)). In the N-group LBM increased from 53.9 kg (95% CI: 51.1–56.7) at baseline to 54.7 kg (95% CI: 51.9–57.4) at week 24 (*p* = 0.003). The LBM increase was significant and similar in the two sex-groups for the N-group, but only for females in the J-group (Table 2). No significant difference between the J- and N-group was detected.

VO₂ max increased in both cheese groups during the 24-weeks trial period (Figure 2(d)). In the J-group VO₂ max increased from 63.1 ml/kg/min (95% CI: 59.8–66.4) at baseline to 65.8 ml/kg/min (95% CI: 62.3–69.3) at week 24 (*p* < 0.001). In the N-group the increase from 63.6 ml/kg/min (95% CI: 60.3–66.8) to 66.8 ml/kg/min (95% CI: 63.5–70.1) (*p* < 0.001). VO₂-max increased for female and males in both cheese groups, but no significant differences between sexes were detected (Table 2). The Testosterone level tended to increase by 1.9 nmol/L in the J-group and tended to decrease slightly by 0.2 nmol/L in the N-group. None of these changes were significant and no significant difference between groups was detected (Table 2).

**Figure 3.**
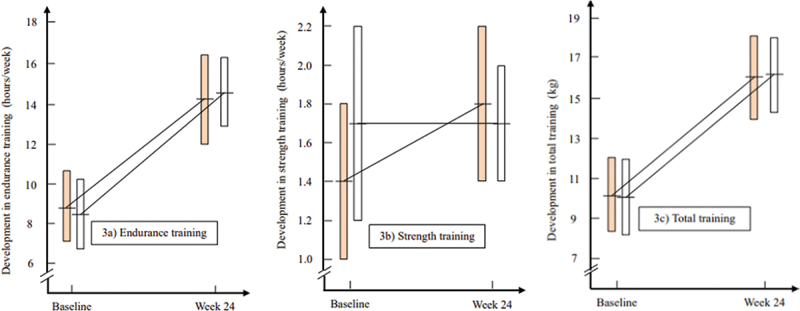
Development in a) Endurance training, b) Strength training, c) Total training from baseline to week-24. The columns express 95% confidence intervals of the mean values indicated by the horizontals line crossing the orange columns 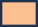 for the Jarlsberg group and white columns for 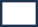 Norvegia.

The duration of endurance training showed a significant increase from baseline to 24 weeks in both cheese groups for both genders (Figure 3(a)), with no notable differences observed between the groups (Table 3). Additionally, the hours of strength training increased significantly in the J-group, whereas no changes were detected in the N-group (Figure 3(b)), and no significant difference between the groups was identified. Of notice, strength training was characterized by muscular endurance training, and to a lesser extent, maximal strength training. Total training increased significantly (*p* < 0.01) in both groups (Figure 3c) and no differences between groups were detected (Table 3). The energy intake increased significantly both in the cheese- and the sex groups from baseline to 24 weeks and no differences between groups were found (Table 3).

**Table 3.** Comparison of Jarlsberg® and Norvegia cheese in development of training, osteocalcin and energy intake during 24 weeks of daily cheese intake. The total number of participants in the Jarlsberg and the Norvegia cheese groups are given on the table. The results are expressed by means with SD in brackets and 95% confidence intervals.

Variables	Sex	Baseline		Week 24		Increase from baseline		P-values
		Jarlsberg	Norvegia	Jarlsberg	Norvegia	Jarlsberg	Norvegia	
Endurance Training (hours/week)	Female (*n* = 25)	n = 12	n = 13	n = 12	n = 13	n = 12	n = 13	p = 0.84
		9.3 (4.2)	9.4 (5.2)	13.6 (4.1)	13.7 (4.4)	4.3 (4.1)	4.4 (4.3)	
		6.6–11.9	6.2–12.5	11.0–16.2	11.1–16.4	1.8–6.9	1.8–7.0	
	Male (*n* = 25)	n = 13	n = 12	n = 13	n = 12	n = 13	n = 12	p = 0.76
		8.4 (4.7)	7.5 (2.9)	14.7 (6.2)	15.2 (4.1)	6.4 (7.4)	7.7 (5.3)	
		5.5–11.2	5.6–9.3	11.0–18.4	12.6–17.8	1.9–10.8	4.3–11.1	
Strength Training (hours/week)	Female (*n* = 25)	n = 12	n = 13	n = 12	n = 13	n = 12	n = 13	p = 0.42
		1.1 (0.6)	1.7 (1.1)	1.4 (0.6)	1.7 (0.9)	0.3 (0.7)	0.0 (0.5)	
		0.7–1.5	1.0–2.4	1.1–1.8	1.2–2.3	−0.1–0.8	−0.3–0.4	
	Male (*n* = 25)	n = 13	n = 12	n = 13	n = 12	n = 13	n = 12	p = 0.36
		1.7 (1.1)	1.6 (1.4)	2.1 (1.0)	1.7 (0.7)	0.4 (0.9)	0.0 (1.3)	
		1.0–2.4	0.8–2.5	1.5–2.7	1.2–2.1	−0.2–0.9	−0.8–0.8	
Total Training (hours/week)	Female (*n* = 25)	n = 12	n = 13	n = 12	n = 13	n = 12	n = 13	p = 0.82
		10.4 (4.4)	11.1 (5.5)	15.1 (4.3)	15.5 (4.5)	4.7 (4.3)	4.4 (4.4)	
		7.6–13.1	7.8–14.4	12.3–17.8	12.8–18.2	1.9–7.4	1.8–7.1	
	Male (*n* = 25)	n = 13	n = 12	n = 13	n = 12	n = 13	n = 12	p = 0.68
		10.1 (4.9)	9.1 (3.7)	16.8 (5.5)	16.8 (4.2)	6.7 (7.5)	7.7 (5.7)	
		7.1–13.0	6.8–11.4	13.5–20.1	14.7–19.5	2.2–11.3	4.1–11.4	
Total Osteocalcin (tOC) ng/ml	Total (*n* = 58)	n = 29	n = 29	n = 29	n = 29	n = 29	n = 29	p < 0.01
		39.4 (16.2)	38.4(15.9)	38.0(17.5)	26.8(7.6)	−1.4(9.1)	−11.6(10.3)	
		33.2–45.5	32.4–44.4	31.3–44.6	23.9–29.7	−4.9–2.1	−15.5- −7.7	
Carboxylated Osteocalcin (cOC). ng/ml	Total (*n* = 58)	n = 29	n = 29	n = 29	n = 29	n = 29	n = 29	p < 0.01
		22.0 (8.9)	19.5(6.7)	23.3(14.1)	16.3(4.2)	1.3(6.5)	−3.2(4.0)	
		18.7–25.4	17.0–22.1	17.2–33.5	15.6–20.3	−1.2–3.8	−4.7- −1.7	
P1NP/CTX-ratio	Total (*n* = 58)	n = 29	n = 29	n = 28	n = 29	n = 28	n = 29	p = 0.05
		158(49.6)	195 (61.2)	175 (47.8)	194 (67.0)	19.2 (48.7)	−0.7 (44.9)	
		139–177	172–218	156–194	169–220	0.4–38.1	−17.8–16.4	
Energy intake (kcal)	Total (*n* = 56)	n = 27	n = 29	n = 27	n = 29	n = 27	n = 29	p = 0.93
		2072 (792)	2656 (785)	2897 (699)	3268 (879)	624 (501)	611 (635)	
		1959–2596	2357–2955	2620–3173	2933 – 3602	426–822	370–853	

Both tOC and cOC decreased significantly (*p* < 0.04) during the 24-week trial period in the N-group but no changes were detected in the J-group (Table 3). The OC-levels were significantly (*p* < 0.01) higher in the J-group compared to the N-group at the end of the study. The P1NP/CTX bone-marker ratio significantly increased in J-group but remained stable in the N-group. The increase in the ratio was significantly (*p* = 0.04) larger in the J-group (Table 3).

**Figure 4.**
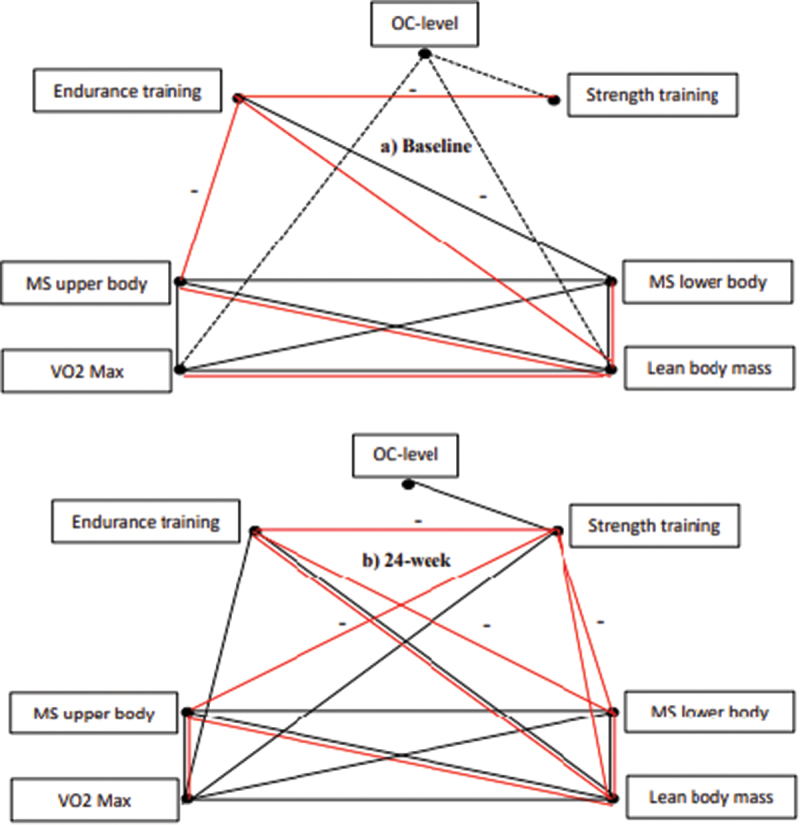
The correlation pattern between and within the outcome variable (MS_ub_, MS_lb,_ LM, VO_2_ Max) and the input variables (training, OC-level) at a) baseline and b) 24-weeks. The black lines show significant multiple Pearson correlations and the red show partial correlations. Dotted black lines show borderline significant (0.05 < p ≤ 0.10) correlations.

The four outcome variables were all significantly and positively multiple correlated both at baseline and after 24 weeks (Table 4). At baseline the correlations between MS_ub_ and VO₂ max, MS_ub_ and MS_lb_ and VO₂ max and MS_lb_ disappear in the partial correlation analyses and are explained by LBM (Figure 4(a)). By including the input variables, endurance training was partially and significantly positively correlated to LBM and OC-level multiple positively correlated to LBM. However, the correlation between OC levels and LBM, as well as between OC levels and VO_2_-max, partially disappeared in the next step of the analysis, and was explained by the training variables. The dominant outcome variable at baseline is LBM and the dominant input variable seems to be endurance training. Endurance training and OC-level along with sex explained 65% of the LBM variation.

**Table 4.** Multiple- and partial correlation coefficients between muscle strength, lean body mass, VO_2_ Max, total osteocalcin level, aerobic- and strength exercise at baseline and 24 weeks. The Pearson correlation coefficients (black) and partial correlation coefficients (red) are given horizontally below the table-diagonal for baseline and above for 24 weeks. Significant correlation coefficients are given in bold.

Sex	24 weeks							
Baseline	Variables recorded on baseline	MS upper body (*n* = 57)	MS Lower body (*n* = 47)	Lean body mass (*n* = 56)	VO₂ - Max (*n* = 53)	Total Osteocalcin (*n* = 58)	Training	
							Endurance (*n* = 50)	Strength (*n* = 50)
**Baseline**	Muscle strength Upper body (*n* = 57)	**1**	**0.65**	**0.85**	**0.65**	0.01	0.22	−0.02
			0.02	**0.68**	**0.27**	0.12	−0.20	**−0.27**
	Muscle strength Lower body (*n* = 47)	**0.66**	**1**	**0.70**	**0.45**	−0.05	−0.05	−0.08
		−0.01		**0.46**	**−0.08**	0.01	**−0.34**	**−0.29**
	Lean body mass (*n* = 56)	**0.83**	**0.66**	**1**	**0.67**	0.03	**0.27**	0.10
		**0.70**	**0.45**		0.21	−0.16	**0.32**	0.41
	VO^2^ (*n* = 53)	**0.57**	**0.43**	**0.66**	**1**	0.01	**0.32**	−0.09
		0.08	−0.11	**0.36**		0.11	0.15	−0.06
	Total Osteocalcin (*n* = 58)	0.19	−0.02	0.21	0.18	1	−0.05	**0.25**
		0.10	−0.05	−0.03	0.06		−0.14	0.14
	Endurance training (*n* = 50)	−0.21	**−0.33**	−0.01	−019	0.13	**1**	−0.21
		**−0.48**	−0.10	**0.52**	−0.22	0.12		**−0.45**
	Strength training (*n* = 50)	−0.01	−0.02	0.10	−0.10	0.19	0.18	**1**
		−0.11	0.13	0.16	−0.17	0.18	0.02	

At week 24, the correlation pattern within the outcome variables was nearly as described for baseline. Correcting for MS_ub_, in the partial correlation analysis, the correlation between LM and VO₂-max disappears and is explained by MS_ub_ (Figure 4(b)). Similarly, the correlation between MS_ub_ and MS_lb_ and between VO₂ max and MS_lb_ disappears when correcting for LBM. The dominant week-24 outcome variable seems to be LBM but supported by MS_ub_.

Including the input variables in the week-24 pattern, endurance training was significantly, positively multiple correlated to LBM and VO₂ max, and strength training to VO₂ max and OC-level (Figure 4(b)). In the partial correlation analysis, these correlations disappear except for the correlation between endurance training and LBM. LBM as outcome variable and endurance training as input variable seem to be the dominant variables in the 24-week pattern. In addition to sex, endurance training and OC-level explains 68% of the variation in LBM.

## 4. Discussion

The relationship between OC and muscle strength, along with Pf’s potential impact on athletes` endurance, prompted our interest in exploring the effects of cheese on MS, LM and VO_2_ max in Nordic skiers [3–5].

### 4.1. Muscle strength and lean mass

Both MS_ub,_ MS_lb_ and LBM increased significantly in both cheese groups, but no significant differences between the groups were detected. There was a trend in the female J-group to increase more than the female N-group both in MSub and LBM. Neither MS nor LBM differentiated in development between the cheese groups. To our knowledge, only cross- sectional studies have shown associations between OC-level and exercise volume, and age and OC-level, but no controlled, prospective clinical studies have examined the effects of stimulating OC. This intervention study found no evidence that J is superior to N in promoting MS and muscle mass development. Although OC development in J-group was significantly higher than in the N-group, this does not appear to influence the development of MS or LBM. To determine whether an 4% better increase in the female J-group and 2% in the male J-group compared to the N-groups could be defended statistically, the sample size must be doubled. In a performance environment, one will always strive for optimization and peak performance, and it is conceivable that the intake of J could contribute to this, especially for elite athletes. However, the detected differences between groups in LBM and VO_2_ max are not clinically significant. To make these differences statistically significant, the sample size must be increased at least ten-fold. The conclusion is that the groups are similar. The only variable which could be clinically significant is MSub. In some cases, there appear to be trends of sex-specific effects; however, the sample size is too small to determine if these differences are statistically significant. It was not an aim to compare sexes.

There was no difference in development in testosterone between the male groups, suggesting no beneficial effect from stimulated testosterone production in the J-group relative to the N-group.

### 4.2. VO_2_ max

The VO_2_ max significantly increased in both cheese and sex groups, but there were no differences between any of the groups. VO_2_ max was selected as a performance variable to assess the potential effects of Pf and propionic acid on performance. A murine study on propionic acid bacteria (PAB) demonstrated an increase in running time in mice during treadmill tests [17]. This finding was suggested to result from lactic acid removal from the intestine by PAB. Through this mechanism, it was hypothesized that the gut could function as a metabolic sink, where lactate diffuses from the bloodstream into the gut, removed by transformation to propionate, potentially reduces serum lactate levels [17]. This could allow performance to continue without the detrimental effects of lactate accumulation [17]. The propionic acid produced in this process can be absorbed from the gut and serve as an energy substrate. These effects are independent of training-induced changes that enhance VO_2_ max by increasing the heart’s stroke volume and oxygen utilization rate [17]. In the present study on Nordic Skiers no such effects were detected. We think that the doses of Pf were sufficient. An additional performance measure like “time to exhaustion” which was used in the murine studies, and a longer study duration could capture functional changes in a better way.

### 4.3. What influences the increased MS and VO_2_ max?

To examine the baseline status in relation to outcome variables and training conditions, a multiple and partial correlation analysis was conducted on the entire study sample. Differences in training experience causes differences in muscle strength, aerobic endurance and LM. Endurance training is the dominant input variable, and lean mass is the dominant outcome variable, explaining both MS_ub_, MS_lb_, and VO_2_ max. The dominant input variable is low at baseline, but there is a carry-over effect from previous training history when examining the outcome variables. When the correlation analysis is repeated at 24 weeks, the same pattern in outcome variables is observed. There remains a carry-over effect from previous training experience. However, the increase in endurance and strength training during the study has made the correlation between endurance and strength training and MS_ub_ more evident. Our results indicate that lean mass is more related to endurance training and less pronounced as a result of strength training. LBM improves both MS_ub_ and MS_lb_. It is possible that certain types of endurance training, such as roller skiing with much double poling, cycling, and running, may provide adequate strength-promoting stimuli for muscle tissue [21,36]. Additionally, VO_2_ max is influenced by endurance training through lean mass and MS_ub_. Our results indicate that LBM is an important predictor of VO_2_ max. In contradiction to the restrictive eating behavior and weight control often observed among skiers, our results indicate that LBM is more important for performance. Therefore, skiers should focus on optimizing training activity combinations and refueling strategies to enhance muscle recovery and promote hypertrophy.

At baseline, there is a borderline significant (*p* = 0.07) correlation between OC, LM and VO_2_ max and strength training. These correlations are explained by the correlation patterns within the outcome variables. At week 24, OC is explained by the correlation between endurance training and LBM. OC seems to be linked to endurance training and LBM, but correlations between OC, MS and LBM are doubtable. It is known that OC-levels are high in adolescents and athletes [37,38]. Studies have shown correlations between higher osteocalcin (OC) levels and maintained muscular strength during weight loss in obese men. Additionally, there is a correlation between good skeletal muscle mass and elevated OC levels in postmenopausal women. These findings are likely influenced by the higher training levels in individuals with elevated OC levels, rather than the OC itself [4,5,10]. The correlation pattern suggests that the impact on LBM seems to be more explained by endurance training than OC. MSub tends to develop more in the J-group than in the N-group, and OC-levels remained elevated in the J-group. A larger sample would help determine if increasing OC leads to increased MSub.

### 4.4. Strengths and limitations

The strength of the study lies in its randomization and prospective longitudinal design. To accurately assess the effects on MS, LBM, and VO_2_ max, a training intervention should have been conducted, involving the provision of different cheeses to a training group and a control group that did not undergo training. Additionally, we should have monitored changes in performance using various measures beyond VO_2_ max, such as “time to exhaustion,” like in the murine experiments, at a standardized speed and incline on the treadmill. There are certain limitations regarding self-report of training load and cheese compliance. The lack of blinding could give some contamination bias, and the sample was too small to sort out differences by sex. A major limitation of this study is that the *Propionibacterium freudenreichii* content of the cheese was not directly measured; therefore, the exact amount is unknown, and the dose was estimated based on historic analytical results. The optimal daily dose determined on average prior to studies on athletes cannot be directly transferred to the athletes, and we also had to account for any sex-specific differences. A potential future dose-response study could aim to determine the cheese dose relative to weight. The study material would then need to be quintupled per gender, as we would have to divide into a minimum of five weight classes for both genders and conduct a dose-response study for each weight class and sex.

## 5. Conclusion

There were no differences in MS, LBM, and VO2-max development between N and J. The significant increase in these metrics is likely due to increased endurance training volume and intensity during the off-season. Training that increases LBM is crucial for improving MS and VO2-max. No direct effect from Pf or increased OC-level was detected, although OC contributes to explaining LBM at baseline and week 24. Given the lack of group differences, consumption of PF-rich cheese did not yield any additional improvements in sport performance.

## Data Availability

The data that support the findings of this study are available from the corresponding author, HEL, upon reasonable request.

## Protocol

The protocol, XCS-Jarlsberg/IIA is registered and can be accessed in trial registry ClinicalTrial.gov: NCT06688032.
